# Educational determinants of immunization coverage among internally displaced persons (IDPs) in Mogadishu: a cross-sectional study

**DOI:** 10.1186/s13690-025-01707-z

**Published:** 2025-09-01

**Authors:** Yusuf Hared Abdi, Yakub Burhan Abdullahi, Mohamed Sharif Abdi, Sharmake Gaiye Bashir, Naima Ibrahim Ahmed

**Affiliations:** 1 Department of Environmental Science, Faculty of Geoscience & Environment, Hormuud Univeristy, Mogadishu, Somalia; 2 De Martino Public Hospital, Ministry of Health and Human Services, Federal Government of Somalia, Mogadishu, Somalia

**Keywords:** Immunization, Vaccine attitudes, Coverage, Internally displaced persons

## Abstract

**Background:**

Immunization remains a cornerstone of global public health; however, Somalia faces critical challenges in achieving equitable vaccination coverage, particularly among internally displaced individuals (IDPs). The National immunization rates for diseases such as diphtheria-tetanus-pertussis (DTP3), measles, and polio remain below 50%, exacerbated by decades of conflict, fragile healthcare infrastructure, and socioeconomic disparities. IDPs in Somalia encounter unique barriers, including overcrowded living conditions and limited access to healthcare and mobility, which disrupt care continuity. This study examined the immunization coverage disparities between IDPs and urban residents in Somalia, focusing on the sociodemographic and attitudinal determinants of vaccine uptake. By analyzing factors such as education, income, marital status, and vaccine perceptions, this study aimed to inform targeted strategies to improve vaccination access in conflict-affected settings.

**Method:**

A cross-sectional study was conducted in March 2025 across two IDP camps (ANFAC and Sahal) in Somalia’s Banadir Region. Using stratified systematic sampling, 384 participants were enrolled, and data were collected via structured questionnaires administered in Somali. Vaccination status was verified through immunization cards or self-reports, and the predictor variables included age, education, occupation, income, marital status, and attitudes toward vaccine safety and efficacy. Statistical analysis employed Chi-square tests and multivariate logistic regression were used to identify the determinants of vaccine uptake with adjustments for confounders.

**Result:**

This study revealed significant immunization disparities primarily associated with educational attainment. Participants with secondary education achieved vaccination rates of 72.6% versus 41.2% among those without formal education. Multivariate analysis identified secondary education (AOR = 3.82, 95% CI: 1.74–8.40, *p* = 0.001) and tertiary education (AOR = 7.95, 95% CI: 3.33–19.01, *p* < 0.001) as the strongest predictors of full vaccination, followed by marital status (divorced/widowed: AOR = 0.33, 95% CI: 0.14–0.81, *p* = 0.015). Household income and positive vaccine attitudes showed no significant association in the adjusted model.

**Conclusion:**

Educational disparities emerge as the most critical barrier to immunization among Somali IDPs. The findings highlight the need for integrated interventions prioritizing community-led education programs and mobile vaccination clinics with cold chain capacity. These strategies, combined with health system strengthening for mobile populations, could reduce zero-dose children by 50% and advance Immunization Agenda 2030 targets. The study underscores that improving access to education may have greater impact on vaccine uptake than economic interventions alone.

**Table:** 

Text box 1. Contributions to the literature
• First comprehensive analysis of vaccination disparities among Somalia’s internally displaced persons, identifying education as the strongest predictor of vaccine uptake.
• Demonstrates that household income does not independently predict vaccination when education is considered, challenging purely economic intervention approaches.
• Establishes that mobile vaccination with community education may be more effective than income-based interventions for reaching zero-dose children.
• Provides baseline coverage data informing Immunization Agenda 2030 equity targets and evidence-based recommendations for health system strengthening in fragile settings.

## Introduction

Immunization is one of the most cost-effective public health interventions for reducing childhood morbidity and mortality worldwide, preventing an estimated 2–3 million deaths annually from vaccine-preventable disease [1]. The global health community has established ambitious targets through successive strategic frameworks, beginning with the Global Vaccine Action Plan (GVAP) 2011–2020, which called for all countries to achieve at least 90% national coverage of routine vaccines by 2020 [2]. This commitment was further reinforced through the Immunization Agenda 2030 (IA2030) endorsed by the World Health Assembly in 2020, which envisions a world where everyone, everywhere, at every age, fully benefits from vaccines [3, 4]. The IA2030 strategy specifically aims to reduce the number of zero-dose children those who have not received even the first dose of diphtheria-tetanus-pertussis-containing vaccine by 50% and to achieve equitable vaccination coverage globally [3]. Despite these global initiatives and the demonstrated effectiveness of vaccination programs, significant challenges persist in achieving universal coverage, with 25.0 million infants worldwide remaining unvaccinated with the third dose of diphtheria-tetanus-pertussis-containing vaccine (DTPcv3) in 2021, representing 19% of the target population [5]. The importance of maintaining high immunization rates extends beyond individual protection to encompass community-wide benefits through herd immunity, thus making vaccination coverage a critical indicator of health system performance and population health resilience [4].

Somalia presents a particularly challenging context for immunization delivery, with national vaccination coverage rates far below global targets. According to recent estimates, coverage for key antigens such as DTP3, measles, and polio remains under 50%, and full immunization among children is as low as 20% [6, 7]. These figures underscore the magnitude of gaps in routine immunization services across the country. Compounding the issue are Somalia’s fragile health system and decades of conflict and instability, which continue to disrupt vaccine access and erode public trust [8]. However, recent efforts have demonstrated the potential for improvement through context-specific strategies, as evidenced by Somalia’s COVID-19 vaccination campaign, which achieved 42.1% coverage by December 2022 despite initial challenges, including inexperience in managing mass adult vaccination, inadequate infrastructure, and health workforce limitations [8]. These campaigns also yielded additional benefits, with 84,600 zero-dose children receiving their first childhood vaccine during integrated vaccination activities [8]. Within Somalia’s complex epidemiological landscape, internally displaced individuals represent one of the most vulnerable populations with respect to vaccination access and coverage. IDPs face unique challenges that distinguish their vaccination experiences from those of settled urban populations, including precarious living conditions in overcrowded camps, limited access to healthcare services, and frequent mobility that disrupts the continuity of care [9]. Studies have documented that IDPs consistently demonstrate among the lowest vaccination coverage rates in Somalia, with multiple barriers operating at the individual, interpersonal, community, organizational, and policy levels [9]. The camp environment itself presents specific challenges, including vaccine stock-outs, long waiting times at health facilities, language barriers between healthcare providers and displaced populations, and hesitancy among health workers to open multidose vials for small numbers of children [9].

Previous research has identified multiple determinants that influence vaccination uptake in Somalia and similar conflict-affected settings operating across individual, household, community, and health system levels. At the individual level, maternal education has consistently emerged as a significant predictor of immunization coverage, with higher educational attainment being associated with an increased likelihood of complete vaccination [6]. The place of delivery also plays a crucial role, with facility-based births significantly associated with higher immunization rates than home deliveries [6]. Geographic factors, particularly the distance to health facilities, create substantial barriers to vaccination access, especially for rural and displaced populations [6]. Socioeconomic determinants include household income levels and opportunity costs associated with seeking vaccination services, which can be particularly burdensome for families facing economic hardships [9]. Attitudinal factors represent another critical domain, with perceptions of vaccine safety and effectiveness strongly influencing uptake decisions [10]. Studies in Somalia have documented that having good perceptions about vaccines are associated with significantly higher odds of complete immunization (AOR = 4.976, 95% CI = 2.183–11.340) [10]. However, vaccine hesitancy and refusal remain significant challenges, driven by factors such as low trust in vaccines, misinterpretation of religious beliefs, rumors and misinformation, and Somalia’s patriarchal decision-making structures, where fathers, who are often poorly informed about vaccination, serve as primary decision-makers [11].

This cross-sectional study aimed to determine immunization coverage rates among internally displaced persons (IDPs) and urban residents in Somalia, while assessing sociodemographic and attitudinal predictors of vaccine uptake through multivariate analysis. This study specifically evaluated how awareness levels, perceptions of vaccine safety, and beliefs about effectiveness differ between these vulnerable groups. By analyzing these factors alongside socioeconomic variables, this study addresses critical evidence gaps identified in recent Somali Demographic and Health Survey data to inform targeted vaccination strategies in fragile settings.

## Methodology

### Study area and population

This cross-sectional study was conducted in the Banadir Region of Somalia, focusing on two internally displaced person (IDP) camps, ANFAC and Sahal, both located within the Kahda District. These camps were selected from the official registry to capture variability in camp size and living conditions. Eligible participants were adult residents (aged 18 years and above) who had lived in their respective camps for at least six months and who provided informed consent.

### Study design

A cross-sectional design was employed to assess immunization coverage and related attitudes simultaneously in ANFAC and Sahal, thereby controlling for temporal influences, such as ongoing vaccination campaigns or seasonal movements.

### Sampling strategy and sample size determination

We used a stratified systematic sampling approach, with each camp constituting the stratum. Household lists provided by camp administrators were mapped and every kth household was calculated by dividing the total number of households by the camp’s allocated sample size. Within each selected household, one adult was chosen randomly for the interviews. The overall sample size was calculated using the single-population proportion formula (*p* = 0.50, Z = 1.96, d = 0.05), yielding 384 respondents; this was inflated to 422 to allow for up to 10% non-response. The final allocation to ANFAC and Sahal reflects their relative population sizes.

### Variables and measurements

The primary outcome, vaccination status, was recorded dichotomously: “vaccinated” if the respondent (or their child) had completed the recommended vaccine series verified via immunization card or reliable self-report and “not vaccinated” otherwise. The predictor variables included sociodemographic characteristics (age category, educational level, occupation, income bracket, and marital status), residential stratum (ANFAC vs. Sahal), and vaccine awareness and attitudes (whether the respondent had heard of the vaccine, believed in its effectiveness, and deemed it safe). Attitudinal items were captured as yes/no responses.

### Data collection procedures

Data were collected from March 25 to March 29, 2025. Trained interviewers administered a structured questionnaire developed in English, translated into Somali, and back-translated to ensure fidelity in private settings within the participants’ shelters or nearby community areas.

### Data management and analysis

Questionnaire data were entered into a secure database and analyzed using Stata version 17. Descriptive statistics (frequencies and percentages) summarized participant characteristics and vaccination coverage by camps. Bivariate analyses using chi-square tests were used to compare vaccination status across independent variables, with crude odds ratios and 95% confidence intervals quantifying unadjusted associations. Variables with *p* < 0.20 in bivariate testing were included in a multivariate logistic regression model to identify independent predictors of vaccine uptake; results are presented as adjusted odds ratios with 95% confidence intervals. Model adequacy was assessed using the Hosmer–Lemeshow goodness-of‐fit test, and multicollinearity was checked using variance inflation factors. Statistical significance was set at *P* < 0.05.

To enhance transparency, all bivariate analyses were conducted using chi-square tests, and odds ratios were presented for descriptive comparison. The lack of statistical significance for several variables in multivariate analysis underscores the importance of interpreting findings in the context of adjusted models.

### Ethical considerations

Ethical approval for this study was granted by the Somali National University Institutional Review Board (Ref: JUS/KCC&CT/XT/0009/2025, dated March 20, 2025), and all procedures were conducted in accordance with the Declaration of Helsinki and local regulations; participants provided informed consent prior to enrollment, confidentiality of responses was strictly maintained through anonymized data collection and secure storage, and respondents’ rights to refuse or withdraw at any time without penalty were fully respected.

## Results

Table 1, The presented socio-demographic characteristics of the study population (*n* = 384) reveal a predominantly young cohort, with 61.46% of participants aged ≤ 34 years (29.17% under 25, 32.29% 25–34), reflecting typical displacement demographics where younger individuals constitute the majority. Educational attainment shows moderate dispersion, with 32.29% attaining secondary education and 25.26% lacking formal schooling, suggesting heterogeneous access to education prior to displacement. Economic vulnerability is evident through occupational patterns, as 38.80% identified as housewives and only 20.83% reported formal employment, while income stratification demonstrates 40.10% in the low-income bracket. Marital status data indicate stable family structures (62.50% married) alongside displacement-related social disruption (16.67% divorced/widowed). The urban predominance (71.88%) aligns with the study’s focus on IDP camps within Kahda District’s urban landscape. These baseline characteristics establish critical context for analyzing healthcare access patterns, with the population’s youth, economic constraints, and urban residence likely influencing health-seeking behaviors and vaccine decision-making processes. The demographic heterogeneity underscores the need for stratified interventions addressing distinct subpopulation needs within displaced communities.

**Table 1:** Socio-demographic characteristics of study participants (*n* = 384) in IDP camps, Banadir region, Somalia

Variable	Frequency (*n*)	Percent (%)
**Age Group**		
Under 25	112	29.17
25–34	124	32.29
35–44	98	25.52
45 and above	50	13.02
**Education Level**		
No formal education	97	25.26
Primary	107	27.86
Secondary	124	32.29
Higher education	56	14.58
**Occupation**		
Employed	80	20.83
Housewife	149	38.80
Self-employed	102	26.56
Unemployed	53	13.80
**Monthly Income**		
Low	154	40.10
Medium	144	37.50
High	86	22.40
**Marital Status**		
Single	80	20.83
Married	240	62.50
Divorced/Widowed	64	16.67
**Residence**		
Rural	108	28.13
Urban	276	71.88

**Fig. 1:**
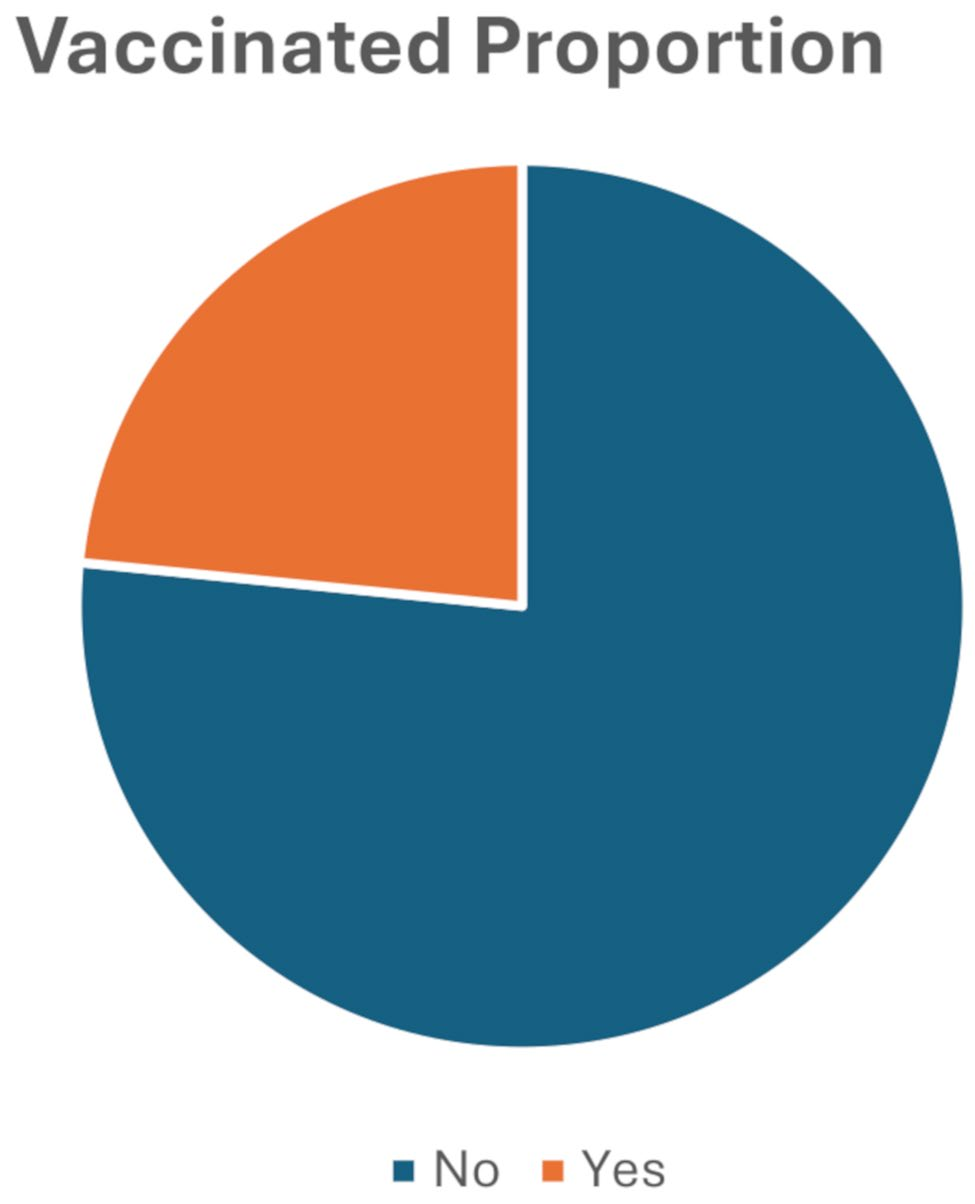
Immunization Coverage Prevalence Among Internally Displaced Persons in ANFAC and Sahal Camps, Banadir Region, Somalia

Figure 1 presents the overall prevalence of complete immunization coverage among the 384 study participants across both the IDP camps. The visualization demonstrates the proportion of individuals who received full vaccination schedules versus those with incomplete or no vaccination coverage, providing a foundational overview of the immunization status within this vulnerable population. The data illustrated in Fig. 1 establish the baseline vaccination rates that informed subsequent bivariate and multivariate analyses examining the sociodemographic and attitudinal determinants of vaccine uptake among internally displaced persons in Somalia’s conflict-affected regions.

Table 2 presents the bivariate analysis of factors associated with immunization coverage among internally displaced persons in the Banadir Region. The analysis reveals that participants with secondary education had a significantly higher vaccination rate (72.6%) compared to those with no formal education (41.2%), and this difference was statistically significant (*p* < 0.001). Similarly, individuals in formal employment reported greater vaccine uptake (68.8%) than housewives (47.0%) and the unemployed (39.6%), with occupation showing a significant association (*p* = 0.002). While income level showed descriptive variation in vaccination rates between high-income (69.8%) and low-income (38.3%) participants, this difference was not statistically significant (medium income *p* = 0.440; high income *p* = 0.721). Similarly, although urban residents had numerically higher coverage (66.3%) than rural residents (44.4%), this difference did not reach statistical significance (*p* = 0.074). Among marital status categories, only divorced/widowed participants showed significantly lower vaccination rates compared to single participants (*p* = 0.023), while married participants did not differ significantly from single participants (*p* = 0.075). General vaccine awareness (*p* = 0.775), beliefs about vaccine prevention capabilities (*p* = 0.471), and beliefs about vaccine safety (*p* = 0.989) showed no significant associations with vaccination status in bivariate analysis. In summary, Table 2 demonstrates that only higher educational attainment (secondary and higher education levels) showed statistically significant associations with improved immunization coverage among the study population. Other factors including income level, urban/rural residence, occupation, and vaccine attitudes did not reach statistical significance in bivariate analysis, with only divorced/widowed marital status showing a significant negative association compared to single participants.

**Table 2:** Bivariate Chi-Square analysis of sociodemographic factors associated with immunization coverage

Variable	Vaccinated	Not Vaccinated	OR [95% CI]	*P*-value
**Age Group**				
Under 25	27	85	1 (ref)	—
25–34	27	97	0.88 [0.48–1.61]	0.670
35–44	26	72	1.14 [0.61–2.12]	0.687
45 and above	10	40	0.79 [0.35–1.78]	0.566
**Education Level**				
No formal education	10	87	1 (ref)	—
Primary	19	88	1.88 [0.83–4.27]	0.132
Secondary	36	88	3.56 [1.66–7.62]	0.001 **
Higher education	25	31	7.02 [3.03–16.25]	0.000 ***
**Occupation**				
Employed	19	61	1 (ref)	—
Housewife	37	112	1.06 [0.56–2.00]	0.856
Self-employed	24	78	0.99 [0.50–1.97]	0.972
Unemployed	10	43	0.75 [0.32–1.76]	0.505
**Income Level**				
Low	39	115	1 (ref)	—
Medium	31	113	0.81 [0.47–1.39]	0.440
High	20	66	0.89 [0.48–1.66]	0.721
**Marital Status**				
Single	26	54	1 (ref)	—
Married	54	186	0.60 [0.35–1.05]	0.075
Divorced/Widowed	10	54	0.38 [0.17–0.87]	0.023 ★
**Residence**				
Rural	32	76	1 (ref)	—
Urban	58	218	0.63 [0.38–1.05]	0.074
**Heard About Vaccines**				
Yes	77	255	1 (ref)	—
No	13	39	1.10 [0.56–2.17]	0.775
**Belief in Prevention**				
Yes	5	23	1 (ref)	—
No	85	271	1.44 [0.53–3.91]	0.471
**Belief in Safety**				
Yes	7	23	1 (ref)	—
No	83	271	1.01 [0.42–2.43]	0.989

Table 3 displays the findings from the multivariate logistic regression analysis assessing independent predictors of vaccination status among children within the study population. After adjusting for potential confounders, household income was not significantly associated with vaccination status (middle income AOR = 0.76, 95% CI: 0.43–1.36, *p* = 0.363; high income AOR = 0.97, 95% CI: 0.50–1.88, *p* = 0.923). In contrast, maternal education showed a strong and significant positive association with vaccination status. Children whose mothers had secondary education (AOR = 3.82, 95% CI: 1.74–8.40, *p* = 0.001) or tertiary education (AOR = 7.95, 95% CI: 3.33–19.01, *p* < 0.001) were substantially more likely to be fully vaccinated compared to those whose mothers had no formal education. Additionally, marital status was associated with vaccination, with divorced or widowed caregivers having lower odds of vaccinating their children (AOR = 0.33, 95% CI: 0.14–0.81, *p* = 0.015). Other variables, including age, occupation, residence, and beliefs about vaccines, did not show statistically significant associations with vaccination status. These results highlight the critical role of maternal education in improving immunization coverage within this internally displaced persons (IDP) context. Interventions targeting educational empowerment may have a greater impact on vaccine uptake than those focused solely on economic factors.

**Table 3:** Adjusted odds ratios (AORs) for predictors of full vaccination status among IDPs in Somalia (Multivariate logistic Regression)

Variable	AOR	95% CI	*P*-value
**Age**			
Age 18–24 (Ref)	1.00	1 (ref)	—
Age 25–34	0.99	0.52–1.92	0.985
Age 35–44	1.47	0.75–2.90	0.262
Age 45+	0.89	0.37–2.15	0.794
**Education**			
No formal education (Ref)	1.00	1 (ref)	—
Primary education	1.86	0.80–4.32	0.146
Secondary education	3.82	1.74–8.40	0.001 **
Tertiary education	7.95	3.33–19.01	< 0.001 **
**Occupation**			
Unemployed (Ref)	1.00	1 (ref)	—
Employed (Govt/Private)	1.22	0.61–2.43	0.566
Business owner	1.47	0.69–3.14	0.316
Other	0.89	0.36–2.24	0.811
**Income**			
Low income (Ref)	1.00	1 (ref)	—
Middle income	0.76	0.43–1.36	0.363
High income	0.97	0.50–1.88	0.923
**Marital Status**			
Single (Ref)	1.00	1 (ref)	—
Married	0.60	0.33–1.08	0.090
Divorced/Widowed	0.33	0.14–0.81	0.015 *
**Residence**			
Urban (Ref)	1.00	1 (ref)	—
Rural	0.63	0.36–1.09	0.100
**Heard about Vaccine**			
No (Ref)	1.00	1 (ref)	—
Yes	1.39	0.66–2.94	0.383
**Belief Vaccine Prevents Disease**			
No (Ref)	1.00	1 (ref)	—
Yes	1.73	0.59–5.06	0.315
**Belief Vaccine is Safe**			
No (Ref)	1.00	1 (ref)	—
Yes	1.13	0.44–2.94	0.796
**Constant**	0.11	0.02–0.63	0.013 *

In summary, higher maternal education levels—specifically secondary and tertiary education were independently associated with substantially increased odds of full vaccination. Marital status also showed a significant association, with divorced or widowed caregivers having lower odds of vaccinating their children. Other variables including household income, employment status, and geographic residence were not statistically significant predictors in the adjusted model.

## Discussions

This study revealed significant disparities in immunization coverage among internally displaced persons in Somalia, with maternal education and marital status emerging as key independent predictors of vaccine uptake. Although employment status, household income, and urban residence appeared associated with vaccination coverage in bivariate analyses, they did not retain statistical significance in the multivariate model, suggesting these effects may be mediated by education or other factors. The findings demonstrated that participants with secondary education achieved substantially higher vaccination rates than those without formal education, Although formal employment and higher income levels appeared associated with improved immunization coverage in the bivariate analysis, these variables did not retain statistical significance in the multivariate model. This suggests that their apparent effects may be mediated or confounded by other variables such as education or marital status [9–11]. Urban residents showed a trend toward higher vaccination coverage than rural counterparts in the bivariate analysis (*p* = 0.074), but this association did not reach statistical significance in the multivariate model. Similarly, although positive vaccine attitudes were significantly associated with coverage in unadjusted analyses, they were not independent predictors after adjustment [6, 7]. Multivariate analysis did not find household income to be an independent predictor of vaccination completion, indicating that economic factors alone may not fully explain immunization disparities in this population [12, 13].

These findings align with Somalia’s broader immunization challenges, where national vaccination coverage remains critically below global targets, with DTP3, measles, and polio coverage estimated at only 42%, 46%, and 47%, respectively [7, 14]. The identified determinants reflect the systemic weaknesses in Somalia’s fragmented health system, which has been severely compromised by decades of conflict and instability, resulting in donor-dependent services with limited financial resources and inadequate vaccine supply chains [15, 16]. The prominence of socioeconomic disadvantage as vaccination barriers is particularly concerning, with effects primarily mediated through educational access rather than direct income effects, given that approximately 60.2% of children in Somalia are classified as zero-dose, with the majority living in conditions similar to those experienced by IDPs [15, 17]. The educational and awareness gaps identified in this study mirror broader patterns documented across Somalia, where patriarchal decision-making structures, misinformation, and limited health literacy contribute to suboptimal immunization outcomes [10, 11, 18]. These results underscore the urgent need for targeted interventions that address both structural barriers and individual-level determinants to achieve the Immunization Agenda 2030 goals of reducing zero-dose children by 50% and ensuring equitable vaccination coverage.

The emergence of education and marital status as strong predictors of immunization uptake reflects Somalia’s complex sociocultural dynamics and systemic health inequality. Higher educational attainment likely enables critical appraisal of vaccine information and navigation of fragmented health systems, consistent with findings from Mogadishu, where maternal education increased full immunization odds 4.98-fold [10]. Marital status may be a proxy for social capital and decision-making autonomy, as married women in Somalia’s patriarchal structure often require spousal approval for healthcare utilization [19]. Divorced/widowed individuals face compounded vulnerabilities from economic precarity and caregiving burdens [20]. The IDP camp environment exacerbates these determinants through overcrowded living conditions that facilitate rapid rumor spread, mobility-disrupting vaccine schedules, and chronic resource shortages, forcing families to prioritize survival over preventive care [9, 21]. Camp residents’ attitudes are further shaped by historical distrust in transient health teams and fear of vaccine side effects without adequate follow-up [19, 21], creating fertile grounds for misinformation that persists despite outreach efforts.

These findings align with regional studies identifying economic barriers as primary vaccination deterrents, mirroring Nigerian IDP camps, where 68% of zero-dose children belonged to households earning <$1.90/day [22]. The urban-rural coverage disparity parallels broader Somali patterns showing 2.3-fold higher DTP3 completion in urban versus rural settings [6], although it contrasts with Ethiopian Somali Region data where pastoralist mobility negated urban advantages [12]. While the strong education-vaccination correlation corroborates Mogadishu’s studies [10], While descriptive differences in vaccination rates were observed across income levels, bivariate analysis did not demonstrate statistically significant income-related vaccination patterns (*p* > 0.05 for all income comparisons). The multivariate analysis confirmed that educational attainment was the primary significant predictor, while income showed no independent association with vaccination status [23]. Notably, the absence of facility distance effects contradicts Ghanaian research, where each kilometer from clinics reduced immunization likelihood by 19% [24], suggesting Somalia’s pervasive access barriers overwhelm proximity advantages.

These findings suggest that favorable vaccine attitudes may be correlated with higher education or income, which themselves predict vaccination uptake, thereby diminishing the independent effect of attitudes after adjustment. This attenuation suggests that the influence of positive vaccine attitudes on immunization coverage may be confounded or mediated by other sociodemographic factors, such as education and household income, which themselves are strong predictors of vaccine uptake. It is possible that individuals with higher education or income are both more likely to hold favorable attitudes toward vaccines and to access immunization services, thereby diminishing the independent effect of attitudes once these variables are controlled for. To explore this possibility, we assessed multicollinearity among the predictor variables using variance inflation factors (VIFs) and found that all VIF values were below the commonly accepted threshold of 5, indicating that multicollinearity was not a significant concern in our model. These findings highlight the complex interplay between socioeconomic status and health beliefs in shaping vaccination behavior among internally displaced populations and underscore the importance of integrated interventions that address both structural barriers and community perceptions.

Closing coverage gaps requires multipronged strategies to address both structural and behavioral determinants. Economic interventions should integrate vaccination conditional cash transfers with existing humanitarian aid distributions, leveraging Somalia’s successful COVID-19 voucher system [25]. Mobile clinics could emulate Somali Red Crescent models, which achieved over 85% measles coverage among nomadic populations through community mobilization and flexible deployment strategies [26]. Meanwhile, decentralized vaccine registries capable of tracking defaulters across IDP camps would help mitigate dropout rates due to population mobility. Additionally, community-led initiatives for training female health volunteers. can improve health literacy, reduce misinformation, and foster trust in immunization services, particularly in displaced settings [27].

### Study limitations

Several limitations warrant consideration. First, the cross-sectional design precludes causal inferences. Vaccination status relied on cards and self-reports (cross-verified via EPI schedules), though recall bias may persist for older vaccinations. Second, the urban focus (two Banadir camps) limits generalizability to rural or nomadic populations, particularly given exclusion of residents with < 6 months’ tenure. Third, despite adjusting for socioeconomic factors, unmeasured sociocultural determinants (e.g., clan affiliation, minority status) may explain residual disparities, necessitating future studies with stratified sampling. Fourth, sample size constraints reduced power for subgroup analyses (e.g., income-education strata). Finally, single-timeframe data collection missed seasonal access variations, while binary vaccination outcomes oversimplified partial immunization patterns. These gaps highlight the need for longitudinal designs with biometric tracking and culturally specific indicators to optimize interventions for mobile populations.

## Conclusion

This study identifies educational attainment and marital status as the primary independent predictors of immunization coverage among Somalia’s internally displaced populations, with secondary and tertiary education increasing vaccination likelihood by 3.8-fold and 8-fold, respectively. In contrast, household income though significant in unadjusted analyses—did not predict uptake after adjustment, underscoring that structural inequities in education and social support systems outweigh purely economic barriers in this fragile setting.

Three key policy priorities emerge: First, community-led education initiatives targeting women and vulnerable groups (e.g., divorced/widowed caregivers) could mitigate knowledge gaps exacerbated by displacement. Second, mobile clinics with cold-chain capacity, modeled after the Somali Red Crescent’s successful nomadic outreach, would improve access for transient populations. Third, integrating vaccination with humanitarian aid distribution (e.g., conditional cash transfers) may optimize resource-limited settings. While positive vaccine attitudes correlated with uptake in bivariate analysis, their attenuation in adjusted models suggests interventions must address structural determinants first to enable behavioral change.

Study limitations including the urban focus, cross-sectional design, and lack of clan/ethnicity data constrain generalizability to rural and pastoralist communities. Future research should employ longitudinal designs and incorporate sociocultural variables (e.g., minority status, language barriers) to better tailor interventions. Nevertheless, these findings provide a roadmap for Somalia to achieve Immunization Agenda 2030’s equity goals. By pairing educational empowerment with health system strengthening, policymakers can reduce zero-dose children among IDPs despite ongoing instability.

## Data Availability

The data supporting the findings of this study are available from the corresponding author upon reasonable request.

## Abbreviations

IDP: Internally Displaced Person
GVAP: Global Vaccine Action Plan
IA2030: Immunization Agenda 2030
AOR: Adjusted Odds Ratio
CI: Confidence Interval
DTP3: Diphtheria-Tetanus-Pertussis third dose
VIF: Variance Inflation Facto

## References

[1] Shrivastava SR, Shrivastava PS, Ramasamy J, WHO. Updates on immunization coverage and how can we improve upon? J Res Med Sci. 2015;20:1216. 10.4103/1735-1995.172992 26958059 10.4103/1735-1995.172992 PMC4766831

[2] Feldstein LR, Mariat S, Gacic-Dobo M, Diallo MS, Conklin LM, Wallace AS. Global routine vaccination coverage, 2016. Morb Mortal Wkly Rep. 2017;66:1252. 10.15585/MMWR.MM6645A3 10.15585/mmwr.mm6645a3 PMC5726243 29145357

[3] Jones CE, Danovaro-Holliday MC, Mwinnyaa G, Gacic-Dobo M, Francis L, Grevendonk J, et al. Routine vaccination Coverage — Worldwide, 2023. Morb Mortal Wkly Rep. 2024;73:978. 10.15585/MMWR.MM7343A4 10.15585/mmwr.mm7343a4 PMC11527360 39480752

[4] O’Brien KL, Lemango E, Nandy R, Lindstrand A. The immunization agenda 2030: A vision of global impact, reaching all, grounded in the realities of a changing world. Vaccine. 2022;42:S1–4. 10.1016/J.VACCINE.2022.02.073 36528445 10.1016/j.vaccine.2022.02.073 PMC9754085

[5] Rachlin A, Danovaro-Holliday MC, Murphy P, Sodha SV, Wallace AS. Routine vaccination Coverage — Worldwide, 2021. Morb Mortal Wkly Rep. 2022;71:1396. 10.15585/MMWR.MM7144A2 10.15585/mmwr.mm7144a2 PMC9639437 36327156

[6] Jama AA. Determinants of complete immunization coverage among children aged 11–24 months in Somalia. Int J Pediatr. 2020;2020:5827074. 10.1155/2020/5827074 32565834 10.1155/2020/5827074 PMC7284922

[7] Belay DB, Ali MI, Chen DG, Jama UA. Prevalence and associated factors of immunization among under-five children in Somalia. BMC Public Health. 2025;25:924. 10.1186/S12889-025-22122-7 40057683 10.1186/s12889-025-22122-7 PMC11889874

[8] Farid M, Ibrahim A, Mohammad H, Hassan Q, Omar MA, Ismael MA, et al. COVID-19 vaccination campaigns in fragile and conflict-affected settings, Somalia. Bull World Health Organ. 2024;102:674. 10.2471/BLT.23.291105 39219761 10.2471/BLT.23.291105 PMC11362698

[9] Jelle M, Seal AJ, Mohamed H, Mohamed H, Omar MS, Mohamed S et al. Understanding multilevel barriers to childhood vaccination uptake among internally displaced populations (IDPs) in mogadishu, somalia: a qualitative study. BMC Public Health 2023;23:2018. 10.1186/S12889-023-16153-1 10.1186/s12889-023-16153-1 PMC10580585 37848917

[10] Hayir TMM, Magan MA, Mohamed LM, Mohamud MA, Muse AA. Barriers for full immunization coverage among under 5 years children in mogadishu, Somalia. J Family Med Prim Care. 2020;9:2664. 10.4103/JFMPC.JFMPC_119_20 32984104 10.4103/jfmpc.jfmpc_119_20 PMC7491846

[11] Abdullahi MF, Stewart Williams J, Sahlèn KG, Bile K, Kinsman J. Factors contributing to the uptake of childhood vaccination in Galkayo district, puntland, Somalia. Glob Health Action. 2020;13:1803543. 10.1080/16549716.2020.1803543 32847489 10.1080/16549716.2020.1803543 PMC7480419

[12] Yadita ZS, Ayehubizu LM. Full immunization coverage and associated factors among children aged 12–23 months in Somali region, Eastern Ethiopia. PLoS ONE. 2021;16:e0260258. 10.1371/JOURNAL.PONE.0260258 34874949 10.1371/journal.pone.0260258 PMC8651113

[13] Hassan MS, Abdulla F, Hossain M. Vaccination coverage and its associated factors among children under-5 in Somalia. BMC Public Health. 2025;25:997. 10.1186/S12889-025-21529-6 40082831 10.1186/s12889-025-21529-6 PMC11907845

[14] Fahmy K, Hasan Q, Sharifuzzaman M, Hutin Y. Analyzing subnational immunization coverage to catch up and reach the unreached in seven High-Priority countries in the Eastern mediterranean region, 2019–2021. Vaccines (Basel). 2024;12:285. 10.3390/VACCINES12030285 38543919 10.3390/vaccines12030285 PMC10975705

[15] Mohamoud SA, Ali-Salad MA, Bile AS, Singh NS, Mahmud AJ, Nor B. Determinants and prevalence of zero-dose children in somalia: analysis of the 2020 health demographic survey data. PLOS Global Public Health 2024;4. 10.1371/journal.pgph.0002612 10.1371/journal.pgph.0002612 PMC11218971 38954718

[16] Ibrahim AM, Hamayoun M, Farid M, Al-Umra U, Shube M, Sumaili K, et al. COVID-19 vaccine acceptance and hesitancy in health care workers in somalia: findings from a fragile country with no previous experience of mass adult immunization. Vaccines (Basel). 2023;11:858. 10.3390/VACCINES11040858 37112770 10.3390/vaccines11040858 PMC10144151

[17] Kahow MH, Halane SA, Ali A, Shah R. Health camp’ model: a unique approach for child vaccination in non-state armed actor controlled, inaccessible geographies in Somalia. Glob Health Action. 2024;17:2391598. 10.1080/16549716.2024.2391598 39175410 10.1080/16549716.2024.2391598 PMC11378116

[18] Aarslew LF, Haas N, Khadka PB. Despite misinformation, low trust, and conflict in somalia, high demand for vaccines and a negative endorsement effect of non-state authorities. Sci Rep. 2023;13:21689. 10.1038/S41598-023-48389-7 38066052 10.1038/s41598-023-48389-7 PMC10709303

[19] Hassan MS, Hossain MM. Determinants of vaccination status among Somali children: evidence from a countrywide cross-sectional survey. BMC Pediatr. 2024;24:837. 10.1186/S12887-024-05334-5 39725967 10.1186/s12887-024-05334-5 PMC11674325

[20] Liu H, Nowak GR, Wang J, Luo Z. A National study of marital status differences in early uptake of COVID-19 vaccine among older Americans. Geriatrics. 2023;8:69. 10.3390/GERIATRICS8040069 37489317 10.3390/geriatrics8040069 PMC10366868

[21] Alawa J, Al-Ali S, Walz L, Wiles E, Harle N, Awale MA, et al. Knowledge and perceptions of COVID-19, prevalence of pre-existing conditions and access to essential resources in Somali IDP camps: a cross-sectional study. BMJ Open. 2021;11:e044411. 10.1136/BMJOPEN-2020-044411 34187818 10.1136/bmjopen-2020-044411 PMC8245279

[22] GAVI. Reaching zero-dose children in Nigeria’s IDP camps 2022. https://www.gavi.org/vaccineswork/reaching-zero-dose-children-nigerias-idp-camps (accessed May 31, 2025).

[23] Hassan SA, Abukar AA, Gutale AS, Hassan AI, Haji AJ, Nur AM, et al. Immunization status and its determinants among children aged 12–23 months at community health centers in mogadishu, somalia: a cross-sectional study. Front Pediatr. 2025;13:1504255. 10.3389/FPED.2025.1504255/BIBTEX 40416437 10.3389/fped.2025.1504255 PMC12101081

[24] Adokiya MN, Baguune B, Ndago JA. Evaluation of immunization coverage and its associated factors among children 12–23 months of age in Techiman municipality, ghana, 2016. Archives Public Health. 2017;75:28. 10.1186/S13690-017-0196-6 10.1186/s13690-017-0196-6 PMC5483840 28652913

[25] World Bank Group. Somalia Inks Vaccine Project to Give 11 Million Citizens Protection from Severe Effects of COVID-19 2021. https://www.worldbank.org/en/news/press-release/2021/09/28/somalia-inks-vaccine-project-to-give-11-million-citizens-protection-from-severe-effects-of-covid-19 (accessed May 31, 2025).

[26] On the road with Somalia’s Red Crescent mobile health clinics | International Committee of the Red Cross. n.d. https://www.icrc.org/en/document/somalia-health-mobile-red-crescent-health-drought-malnutrition-water (accessed May 31, 2025).

[27] WHO EMRO |. Somalia launches electronic immunization registry in latest leap of innovation | News | Somalia site n.d. https://www.emro.who.int/somalia/news/somalia-launches-electronic-immunization-registry-in-latest-leap-of-innovation.html (accessed May 19, 2025).

